# Epigenetic silencing of LncRNA ANRIL enhances liver fibrosis and HSC activation through activating AMPK pathway

**DOI:** 10.1111/jcmm.14987

**Published:** 2020-01-20

**Authors:** Jing‐Jing Yang, Yang Yang, Chong Zhang, Jun Li, Yan Yang

**Affiliations:** ^1^ Department of Pharmacology School of Basic Medical Sciences Anhui Medical University Key Laboratory of Anti‐Inflammatory and Immunopharmacology, Ministry of Education Hefei China; ^2^ Department of Pharmacology The Second Hospital of Anhui Medical University Hefei China; ^3^ Department of Surgical Oncology The Affiliated Suzhou Science & Technology Town Hospital of Nanjing Medical University Suzhou China; ^4^ School of Pharmacy Anhui Medical University Hefei China

**Keywords:** antisense non‐coding RNA in the INK4 locus, DNA methyltransferases 3A, epigenetic, hepatic stellate cell, liver fibrosis

## Abstract

Long non‐coding RNAs (LncRNAs) and DNA methylation are important epigenetic mark play a key role in liver fibrosis. Currently, how DNA methylation and LncRNAs control the hepatic stellate cell (HSC) activation and fibrosis has not yet been fully characterized. Here, we explored the role of antisense non‐coding RNA in the INK4 locus (ANRIL) and DNA methylation in HSC activation and fibrosis. The expression levels of DNA methyltransferases 3A (DNMT3A), ANRIL, α‐Smooth muscle actin (α‐SMA), Type I collagen (Col1A1), adenosine monophosphate‐activated protein kinase (AMPK) and p‐AMPK in rat and human liver fibrosis were detected by immunohistochemistry, qRT‐PCR and Western blotting. Liver tissue histomorphology was examined by haematoxylin and eosin (H&E), Sirius red and Masson staining. HSC was transfected with DNMT3A‐siRNA, over‐expressing ANRIL and down‐regulating ANRIL. Moreover, cell proliferation ability was examined by CCK‐8, MTT and cell cycle assay. Here, our study demonstrated that ANRIL was significantly decreased in activated HSC and liver fibrosis tissues, while Col1A1, α‐SMA and DNMT3A were significantly increased in activated HSC and liver fibrosis tissues. Further, we found that down‐regulating DNMT3A expression leads to inhibition of HSC activation. Reduction in DNMT3A elevated ANRIL expression in activated HSC. Furthermore, we performed the over expression ANRIL suppresses HSC activation and AMPK signalling pathways. In sum, our study found that epigenetic DNMT3A silencing of ANRIL enhances liver fibrosis and HSC activation through activating AMPK pathway. Targeting epigenetic modulators DNMT3A and ANRIL, and offer a novel approach for liver fibrosis therapy.

## Graphical abstract

Overview of the epigenetic silencing of LncRNA ANRIL enhances liver fibrosis and HSC activation through activating AMPK pathway. DNMT3A contributes to down regulation of LncRNA ANRIL, which is removed from its target genes during chronic liver fibrosis. Over expression ANRIL suppresses HSC activation and AMPK signaling pathways. Targeting epigenetic modulators DNMT3A and ANRIL, and offer a novel approach for liver fibrosis therapy.

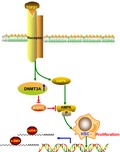

## 1 INTRODUCTION

Hepatic fibrosis is the wound‐healing process in response to chronic liver injury.^1^ Hepatic stellate cell (HSC) constitutes the accumulation of extracellular matrix (ECM) once they activated.^2^ The activated HSC expresses a variety of factors such as transforming growth factor‐β1 (TGF‐β1), which stimulate the HSC activation, and secrete collagens and smooth muscle α‐action (α‐SMA).^3^ This study discusses the molecular and cellular mechanisms of HSC activation and offers a novel approach for liver fibrosis therapy.

Currently, it is known that epigenetic modifications of liver fibrosis‐related genes in liver fibrosis development.^4^, ^5^, ^6^ Epigenetic provides to a heritable modulation in gene expression that does not alter the DNA itself.^7^, ^8^ Epigenetic influences generally refer to aberrant DNA methylation and non‐coding RNA (ncRNA) modifications.^9^, ^10^ With regard to the latter, de novo DNA methylation activity catalysed by DNA methyltransferase 3A (DNMT3A) is methylated by addition of transfer methyl groups to the C‐5 position in the cytosine ring.^11^, ^12^ DNA methylation can establish a docking site for transcriptional repressors to permanent gene silencing.^13^ Long non‐coding RNAs (LncRNAs) are well‐known to interact with components of the epigenetic machinery. LncRNAs are longer than 200 nucleotides, which were protein‐non‐coding genes.^14^ LncRNA (antisense non‐coding RNA in the INK4 locus) ANRIL has been demonstrated to play an important role in fibrosis disease.^15^ However, the molecular mechanisms of LncRNA ANRIL in liver fibrosis remain largely unknown.

Here, we document DNA methylation modification and their regulatory enzyme (DNMT3A) and LncRNA ANRIL that accompany liver fibrosis and HSC activation. DNMT3A silencing of LncRNA ANRIL regulates hepatic stellate cell activation through adenosine monophosphate‐activated protein kinase (AMPK) pathway. Our study provides new understanding of epigenetic changes during liver fibrosis and HSC activation. Our findings suggest that epigenetic DNMT3A silencing of ANRIL enhances liver fibrosis and HSC activation through activating AMPK pathway, and offer a novel approach for liver fibrosis therapy.

## 2 MATERIALS AND METHODS

### 2.1 Reagents

Col1A1, α‐SMA and GAPDH antibodies were obtained from Boster. DNMT3A, TGF‐β1, AMPK and p‐AMPK antibodies were obtained from Abcam. DMSO and MTT assay kit were obtained from Sigma (Sigma‐Aldrich). TGF‐β1 (Peprotech). ANRIL, Col1A1, DNMT3A, α‐SMA and GAPDH primers were purchased by the Shanghai Sangong Company. Secondary antibodies were purchased from Santa Cruz Company.

### 2.2 Animal models of liver fibrosis

Sprague‐Dawley rats (Forty) were obtained from the Anhui Medical University, Experimental Animal Center. SD rats were intraperitoneally injected twice‐weekly for 12 weeks with a mixture of carbon tetrachloride (CCl_4_)/olive oil in a 1:1 (vol/vol) ratio at 1 mL/kg.^16^ All animal experiments were approved by the Institutional Animal Care and Use Committee of Anhui Medical University. Twelve weeks later, the animals were anaesthetized, the rats were then sacrificed and liver tissues collected.

### 2.3 Cell cultures

Hepatic stellate cell‐T6 cell lines were obtained from Shanghai Fumeng Gene Biological Corporation. HSC‐T6 was cultured on plastic in 90% DMEM medium (GIBCO) supplemented with 10% foetal bovine serum (GIBCO), 100 U/mL penicillin sulphate and 100 U/mL streptomycin, in a CO_2_ incubator (Thermo Electronic) with a humidified atmosphere of 5% CO_2_ at 37°C. Cells were treated with TGF‐β1 (10 ng/mL) for 48 hours.

### 2.4 Histological analyses

Human and rat liver tissue sections were prepared, cut at 5 μm and stained with H&E, Sirius red and Masson's trichrome using standard histological techniques. Each section was assessed under light microscopic fields. Semi quantitatively be measured in five randomly selected fields of each liver samples using Image J software.

### 2.5 Immunohistochemistry

Human and rat liver tissue sections were prepared, cut at 5 μm and slides were then permeabilized with 1% saponin/0.5% BSA/PBS for 10 minutes at room temperature, washed, blocked with 3% BSA for 10 minutes and incubated with the one of the following primary antibodies: polyclonal anti‐DNMT3A (1:200), anti‐α‐SMA (1:100), anti‐TGF‐β1 (1:100) and anti‐Col1A1 (1:100). Primary antibodies were detected by rabbit antimouse and goat anti‐rabbit non‐biotinylated regents (Zhongshan). At least five random fields of each section were examined, and semi‐quantitative evaluations were analysed with a Photo and Image Auto analysis System (Image‐Pro Plus 6.0).

### 2.6 Immunofluorescence

Hepatic stellate cells then were fixed in 4% paraformaldehyde, and non‐specific sites were blocked with 10% FBS. Cells then were incubated overnight at 4ºC in primary antibody to detect collagen I, α‐SMA and DNMT3A. After 24 hours of culture, cells were washed with PBS and incubated with TGF‐β1 (10 ng/mL) for 48 hours. After washing, cells were incubated in fluorochrome‐coupled secondary antibody diluted in 1× phosphate‐buffered saline for 1 hour at room temperature. DAPI (Genview Inc) was employed for staining. Finally, fluorescence was visualized with a microscope.

### 2.7 MTT assays

According to the protocol of the manufacturer, cell proliferation assays were carried out by the use of MTT solution. HSC‐T6 (5 × 10^3^/mL) was cultured with concentrations of 10 ng/mL TGF‐β1 for 24, 48 hours in 96‐well plates. According to the requirements of experiments, cell proliferation was observed at 24, 48 hours. Before the observation, MTT (0.5 mg/mL) was helped for incubation of these cells for about three hours at 37°C. After medium was removed, DMSO solution was added into the formazan crystal to dissolve and measure in triplicate at 490 nm wavelength using a Thermomax microplate reader (bio‐tekEL). All experiments were performed in triplicate and repeated at least three times.

### 2.8 CCK‐8 assay

According to the protocol of manufacturer, cell proliferation assays were carried out by the use of CCK‐8 (Dojindo Laboratories). After treatment, cells were seeded in six‐well plates at approximately 2000 per well. After being cultured for 24, 48 hours, cells were added in 10 mL CCK‐8 solutions. After incubating, the OD value was measured at 450 nm wavelengths using a Thermomax microplate reader (bio‐tekEL).

### 2.9 QRT‐PCR

Total RNA was isolated from liver tissues or cells using TRIzol reagent (Life Tech) according to the manufacturer's instruction. To examine the relative expression of DNMT3A, ANRIL, TGF‐β1, α‐SMA and Col1A1, reverse transcription was conducted. Real‐time PCR was performed in 20 μL SYBR Green PCR Master Mix (Takara). Then, GAPDH was viewed as the reference gene to conduct quantitative real‐time PCR with the help of the SYBR Green PCR Master Mix (Takara). Melting curves were put into use for evaluation of non‐specific amplification. The results of qRT‐PCR were converted to fold changes (2^−ΔΔCt^). The sequences of rat primers are listed as following. GAPDH (forward: 5′‐GACATGCCGCCTGGA GAAAC‐3′; reverse: 5′‐AGCCCAGGATGCCCTTTAGT‐3′), α‐SMA: (forward: 5′‐TGGCCACTGCTGCTTCCTCTTCTT‐3′; reverse: 5′‐GG GGCCAGCTTCGTCATACTCCT‐3′), Col1A1: (forward 5′‐TAACTTCTGGAAT TCGACTTTTTGG‐3′; reverse 5′‐GT CCAGCCCTCATCC TGGCC‐3′), TGF‐β1: (forward 5′‐GAGATGCAATGCTATTCCT‐3′; reverse 5′‐CGACGTCACAGCGCAC TT‐3′), DNMT3A: (forward 5′‐CATCCGCCACCTCTTCGCTC‐3′; reverse 5′‐CTCTCCGTCCTCTCGTTCTTGG‐3′), ANRIL: (forward 5′‐TTATGCTTTGCAGC ACACTGG‐3′; reverse 5′‐GTTCTGCCACAGCTTTGATCT‐3′). The sequences of human primers are listed as following. GAPDH (forward: 5′‐ACGAAGCTGAA GCAGGAGAAG‐3′; reverse: 5′‐GGATGAAACCC AGACACATAGC‐3′), α‐SMA: (forward: 5′‐CTGCTGAGCGTGAGATTGTC‐3′; reverse: 5′‐CTCAAGGGAGG ATGAGGATG‐3′), Col1A1: (forward 5′‐GATT CCCTGGACCTAAAGGTGC‐3′; reverse 5′‐AGCCTCTCCATCTTTGCCAGCA‐3′), DNMT3A: (forward 5′‐ACGA TTGCTAGACTGGGATAATG‐3′; reverse 5′‐AGTAAGCAGGCCAGGTAGA‐3′), ANRIL: (forward 5′‐TTGTGAAGCCCAAGTACTGC‐3′; reverse 5′‐TTCACTG TGGAGACGTTGGT‐3′).

### 2.10 Cell transfection

For analysis of the role of DNMT3A and ANRIL, siRNA particularly against DNMT3A and ANRIL and negative control were synthesized by GenePharma Co Ltd. The design and construction of recombinant plasmids, including over expression plasmid pcDNA3.1‐LncRNA ANRIL. For transfection, Lipofectamine™ 2000 (Invitrogen) was used as the transfection reagent in accordance with the instructions of the manufacturer.

### 2.11 Western blotting

Total proteins were extracted from liver tissues and cells. Proteins subjected to 10% SDS‐PAGE for separation, and then they were transferred onto PVDF membranes. Following antibodies were used in this study: antibodies of DNMT3A, α‐SMA, TGF‐β1, Col1A1, AMPK, p‐AMPK and GAPDH were diluted in 1:200‐1:1000. After being blocked using nonfat milk, the membranes were incubated at 4°C with primary antibodies overnight, rinsed with Tris‐buffered saline containing Tween 20 and further incubated with secondary antibody at room temperature for 1 hour. When the washing was finished, chemiluminescence detection kit was employed to examine signals from different proteins.

### 2.12 Statistical analyses

Data were analysed by SPSS17.0 statistical software (SPSS Inc), where measurement data were represented by mean ± SD. Comparison was tested by t test, and comparisons among groups were analysed by one‐way analysis of variance (ANOVA). If *P* < .05, it was considered to be statistically significant.

## 3 RESULTS

### 3.1 Pathological change of CCl_4_‐induced experimental liver fibrosis

**Figure 1:**
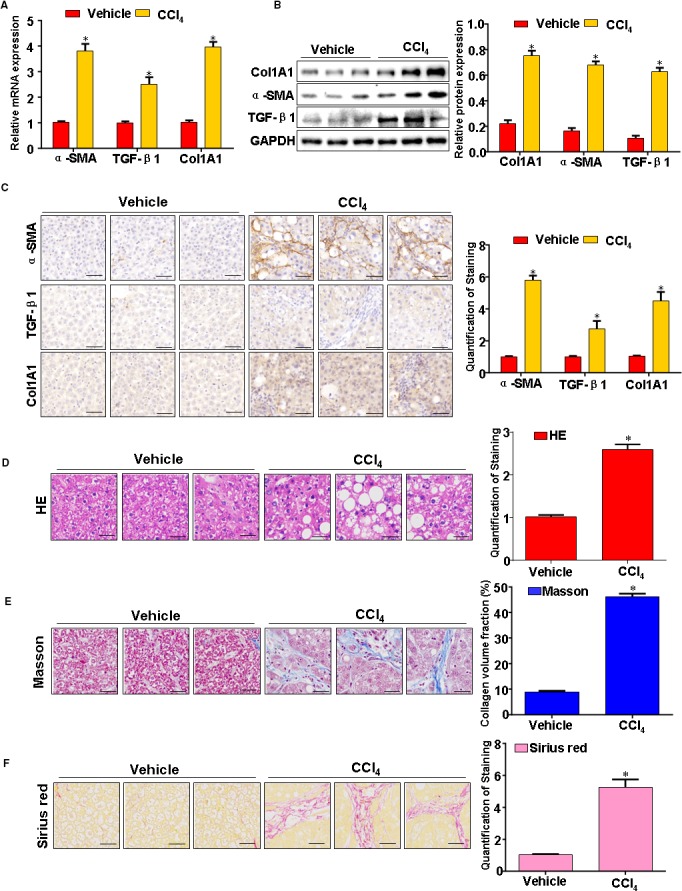
Pathological change in CCl_4_‐caused rat liver fibrosis model. A, The TGF‐β1, α‐SMA and Col1A1 expression were evaluated by qRT‐PCR. B, The TGF‐β1, α‐SMA and Col1A1 expression were evaluated by Western blotting. C, The TGF‐β1, α‐SMA and Col1A1 expression were evaluated by immunohistochemistry. D, Liver fibrosis tissue was fixed with formalin, and then it was embedded in paraffin. Thin sections were cut and stained with haematoxylin & eosin (H&E), Masson's trichrome stain (E) and Sirius red staining (F). Representative views from each group are presented. Data are representative of at least three separate experiments. *P < .05, **P < .01 vs control (vehicle)

To begin to determine whether fibrosis is associated with pathological changes in the liver tissues, we examined the hepatic expression of TGF‐β1, α‐SMA and Col1A1. TGF‐β1, Col1A1 and α‐SMA are increased in liver fibrosis tissues (Figure 1A‐C). H&E staining found inflammatory infiltration, steatosis and fibrosis in the liver fibrosis tissues (Figure 1D). Moreover, Masson's trichrome staining demonstrated that collagen deposition significantly increased in the liver fibrosis tissues (Figure 1E). Furthermore, representative stains for Sirius red confirmed liver fibrosis in response to CCl_4_ (Figure 1F). In sum, 12 weeks after CCl_4_‐treated, increased fibrosis, collagen deposition was observed in liver fibrosis tissues.

### 3.2 Fibrosis liver displays aberrant DNMT3A and ANRIL expression while TGF‐β1 causes the similar alterations in HSC

**Figure 2:**
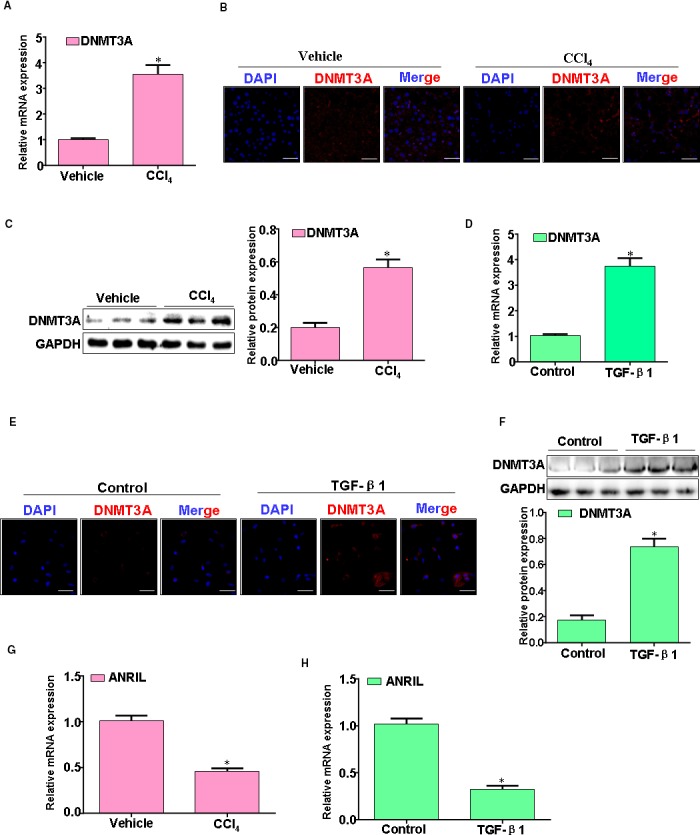
Fibrosis liver displays aberrant DNMT3A and ANRIL expression while TGF‐β1 causes the similar alterations in HSC. A, The DNMT3A mRNA expression was evaluated by qRT‐PCR. B, DNMT3A immunostaining on sections of vehicle rat liver or CCl_4_ rat livers. C, The DNMT3A protein expression was evaluated by Western blotting. D, HSC treatment with TGF‐β1, the DNMT3A mRNA expression was evaluated by qRT‐PCR. E, HSC treatment with TGF‐β1, the DNMT3A protein expression was analysed by immunofluorescence. F, HSC treatment with TGF‐β1, the DNMT3A protein expression was analysed by Western blotting. G, The ANRIL expression was evaluated by qRT‐PCR. H, HSC treatment with TGF‐β1, the ANRIL expression was evaluated by qRT‐PCR. Data are representative of at least three separate experiments. *P < .05, **P < .01 vs control (vehicle)

Given our interest in epigenetic mediators and, specifically, DNA methylation and LncRNA modifications, we examined differences in this domain. DNMT3A proteins and mRNAs were increased in rat fibrosis livers (Figure 2A‐C). We also found that, in particular, DNMT3A was over expressed after TGF‐β1 treatment HSC (Figure 2D‐F). Interestingly, we determined the expression of ANRIL in liver fibrosis tissues. ANRIL expression was significantly down‐regulated when HSC treated with TGF‐β1 in a time‐dependent manner (Figure 2G,H). All together, these results suggest that TGF‐β1 suppresses ANRIL likely associated with aberrant DNMT3A expression in fibrosis liver.

### 3.3 Epigenetic DNMT3A inhibition attenuates TGF‐β1‐dependent HSC activation in vitro

**Figure 3:**
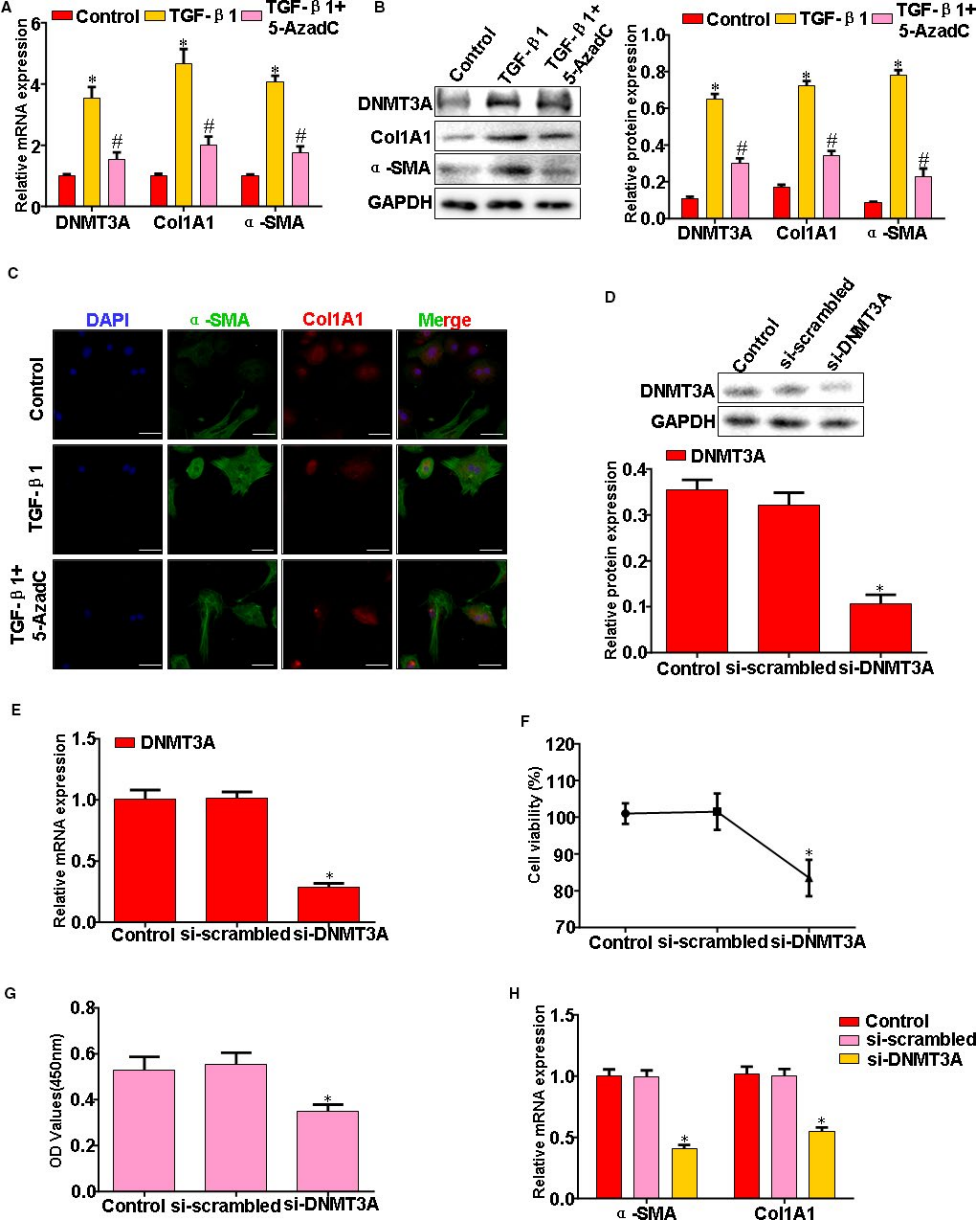
Epigenetic DNMT3A inhibition attenuates TGF‐β1‐dependent HSC activation in vitro. A, HSC treatment with TGF‐β1 and 5‐AzadC, DNMT3A, α‐SMA and Col1A1 expression were analysed by qRT‐PCR. B, HSC treatment with TGF‐β1 and 5‐AzadC, DNMT3A, α‐SMA and Col1A1 expression were analysed by Western blotting. C, HSC treatment with TGF‐β1 and 5‐AzadC, α‐SMA and Col1A1 expression were analysed by Immunofluorescence. D, HSC transfected with si‐DNMT3A or si‐scrambled, DNMT3A expression was analysed by Western blotting. E, HSC transfected with si‐DNMT3A or si‐scrambled, DNMT3A expression was analysed by qRT‐PCR. F, HSC transfected with si‐DNMT3A or si‐scrambled, cell proliferation was measured by MTT assay. G, HSC transfected with si‐DNMT3A or si‐scrambled, cell proliferation was measured by CCK‐8 assay. H, HSC transfected with si‐DNMT3A or si‐scrambled, qRT‐PCR analysis of α‐SMA and Col1A1 mRNA expression. Results are representative of at least three independent experiments. *P < .05, **P < .01 vs Control (Scrambled)

We next sought to examine whether DNMT3A inhibition, either with pharmacologic or genetic approaches, would modulate HSC activation in vitro. Firstly, we used an epigenetic compound, 5‐AzadC (1.0 μmol/L/mL), which specifically inhibitors the catalytic subunit of DNMT3A. Inhibition of DNMT3A in HSC‐treated 5‐AzadC and TGF‐β1 led to a significant decrease in α‐SMA and Col1A1, both at mRNA and protein levels (Figure 3A‐C). To further show the effect of DNMT3A inhibition in HSC, we knocked down the gene using siRNA. HSC transfected with DNMT3A‐siRNA expressed lower levels of DNMT3A relative to cells transfected with a scrambled siRNA (Figure 3D,E). Moreover, treatment of HSC with DNMT3A‐siRNA had a profound inhibitory effect on TGF‐β1‐induced fibroblasts proliferation (Figure 3F,G). Induction of Col1A1 and α‐SMA gene expression is classic events associated with fibroblasts proliferation, both were repressed in DNMT3A‐siRNA‐treated cultures (Figure 3H).

### 3.4 Epigenetic silencing of LncRNA ANRIL is required for HSC proliferation

**Figure 4:**
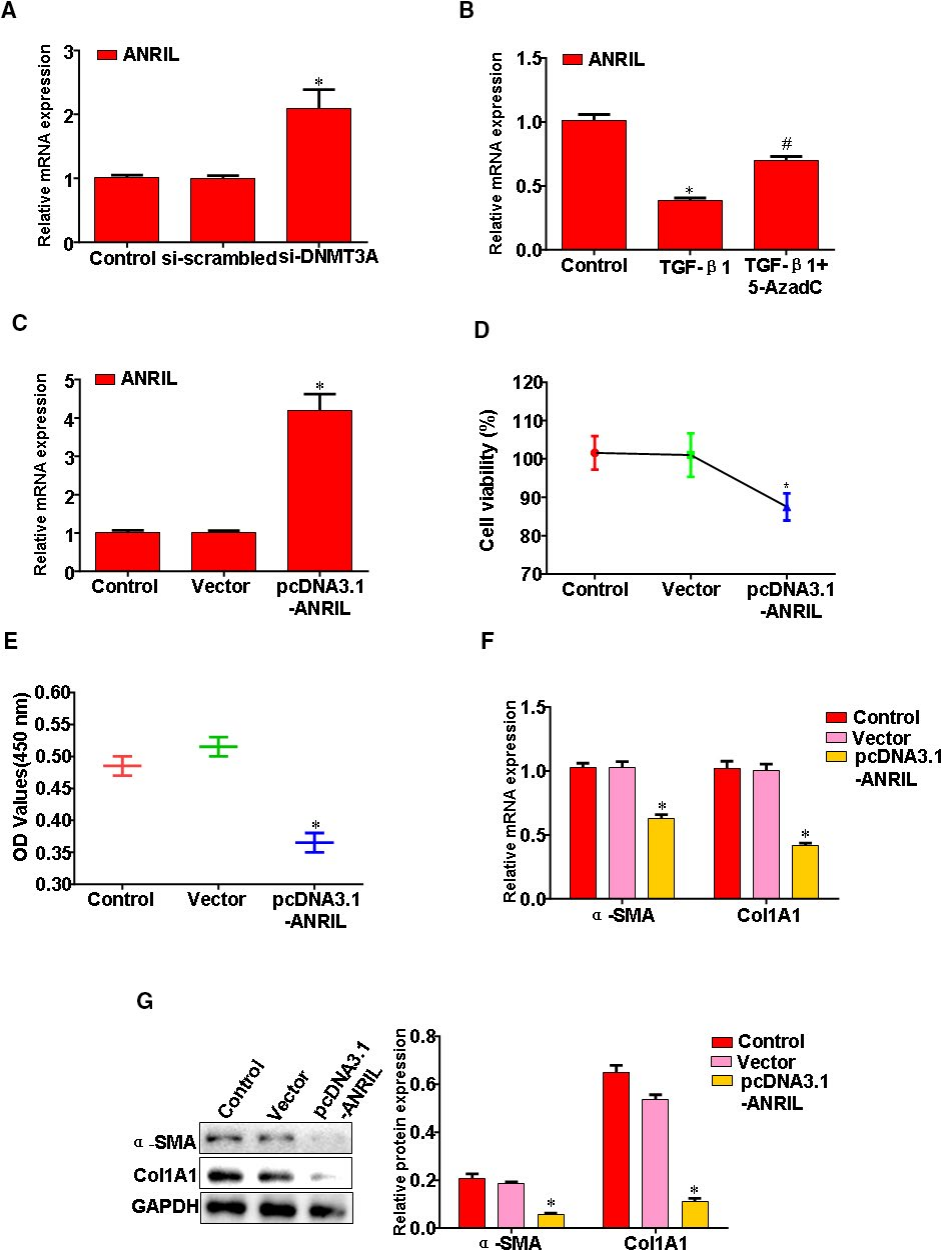
Epigenetic silencing of LncRNA ANRIL is required for HSC proliferation. A, HSC transfected with si‐DNMT3A or si‐scrambled, ANRIL expression was analysed by qRT‐PCR. B, HSC treatment with TGF‐β1 and 5‐AzadC, ANRIL expression was analysed by qRT‐PCR. C, HSC transfected with pcDNA3.1‐ANRIL, ANRIL expression was analysed by qRT‐PCR. D, HSC transfected with pcDNA3.1‐ANRIL, cell proliferation was measured by MTT assay. E, HSC transfected with pcDNA3.1‐ANRIL, cell proliferation was measured by CCK‐8 assay. F, HSC transfected with pcDNA3.1‐ANRIL, α‐SMA and Col1A1 mRNA expression was analysed by qRT‐PCR. G, HSC transfected with pcDNA3.1‐ANRIL, α‐SMA and Col1A1 protein expression was analysed by Western blotting. Data are representative of at least three separate experiments. *P < .05, **P < .01 vs Control or Vector

We were therefore interested to determine whether HSC proliferation is associated with alterations in DNA methylation machinery and DNMT3A marks. HSC transfected with DNMT3A‐siRNA expressed higher levels of LncRNA ANRIL relative to cells transfected with a scrambled siRNA (Figure 4A). Moreover, HSC was treated with 5‐AzadC to promote LncRNA ANRIL expression (Figure 4B). HSC transfected with pcDNA3.1‐ANRIL expressed higher levels of ANRIL relative to cells transfected with a vector (Figure 4C). Furthermore, treatment of HSC with pcDNA3.1‐ANRIL had a profound inhibitory effect on TGF‐β1‐induced HSC proliferation (Figure 4D,E). Induction of Col1A1 and α‐SMA gene expression is classic events associated with fibroblasts proliferation; both were repressed in pcDNA3.1‐ANRIL‐treated cultures (Figure 4F,G).

### 3.5 LncRNA ANRIL depletion triggers HSC activation through AMPK pathway

**Figure 5:**
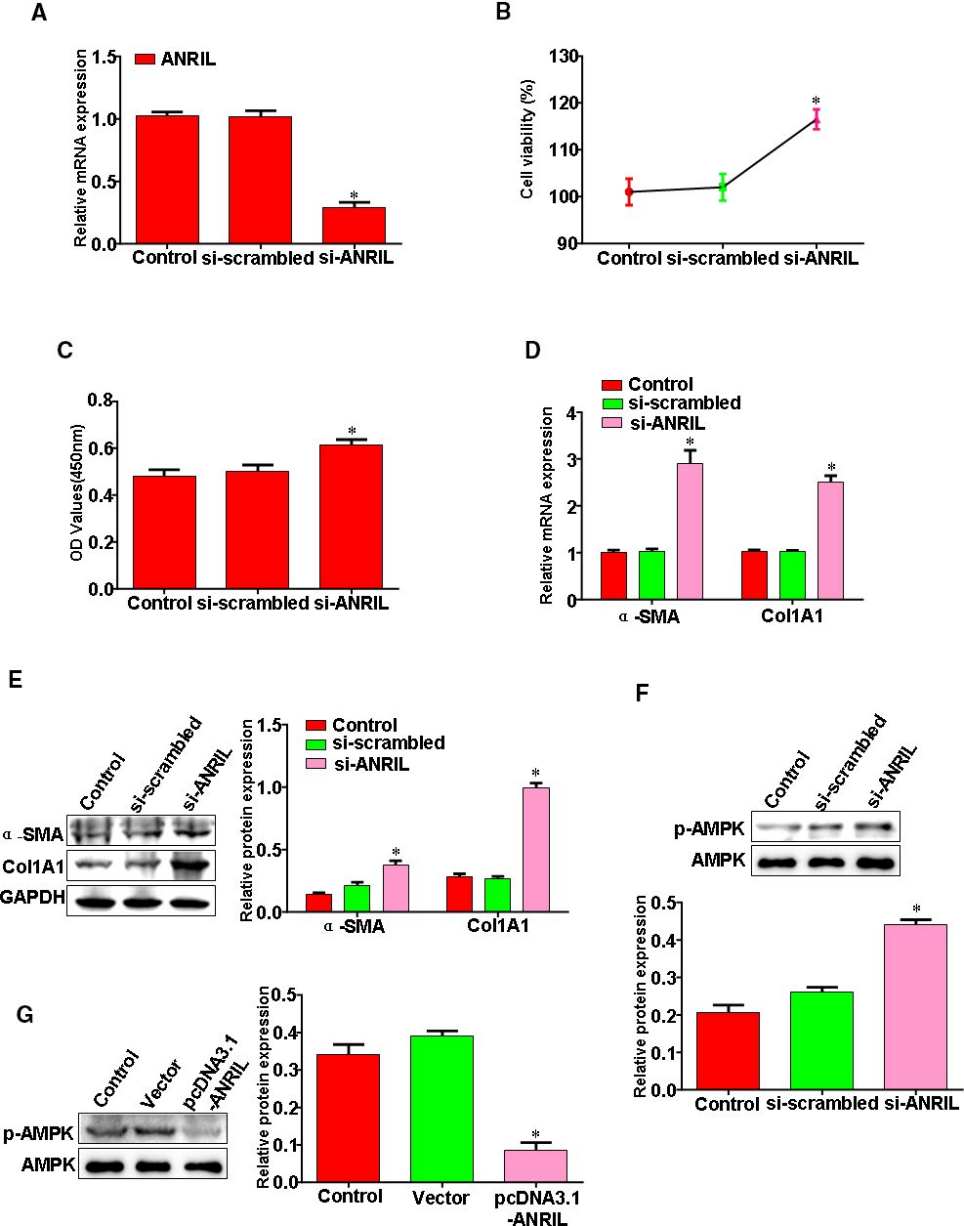
LncRNA ANRIL depletion triggers HSC activation through AMPK pathway. A, HSC transfected with LncRNA ANRIL‐siRNA, ANRIL expression was analysed by qRT‐PCR. B, HSC transfected with LncRNA ANRIL‐siRNA, cell proliferation was determined using MTT assay. C, HSC transfected with LncRNA ANRIL‐siRNA, cell proliferation was determined using CCK‐8 assay. D, HSC transfected with LncRNA ANRIL‐siRNA, α‐SMA and Col1A1 mRNA expression was analysed by qRT‐PCR. E, HSC transfected with LncRNA ANRIL‐siRNA, α‐SMA and Col1A1 protein expression was analysed by Western blotting. F, HSC transfected with LncRNA ANRIL‐siRNA, AMPK and p‐AMPK protein expression was analysed by Western blotting. G, HSC transfected with pcDNA3.1‐ANRIL, AMPK and p‐AMPK protein expression was analysed by Western blotting. Results shown were representative of three independent experiments. *P < .05, **P < .01 vs Control or Vector or Scrambled

To gain the roles of LncRNA ANRIL in regulating HSC activation, we tested the effect of LncRNA ANRIL down‐expression on the HSC proliferation. We found that the expression of LncRNA ANRIL significantly decreased in the HSC transfected with LncRNA ANRIL‐siRNA (Figure 5A). The HSC that was transfected with LncRNA ANRIL‐siRNA had a significantly higher proliferation than NC and vehicle (Figure 5B,C). Moreover, we confirmed the significant down‐regulation of LncRNA ANRIL, which led to a subsequent increase in α‐SMA and Col1A1 (Figure 5D,E). AMPK signal pathway plays a key role in HSC activation. We next wanted to conclusively determine whether AMPK is a key gene in LncRNA ANRIL‐mediated HSC activation. HSC transfected with pcDNA3.1‐ANRIL expressed lower levels of phosphorylated AMPK relative to cells transfected with a vector, while HSC that was transfected with LncRNA ANRIL‐siRNA expressed higher levels of phosphorylated AMPK relative to cells transfected with a scrambled siRNA (Figure 5F,G). These results suggested that LncRNA ANRIL depletion triggers HSC activation through AMPK pathway.

### 3.6 Epigenetic alterations in human chronic liver diseases of distinct aetiologies resemble those associated with rat liver fibrosis

**Figure 6:**
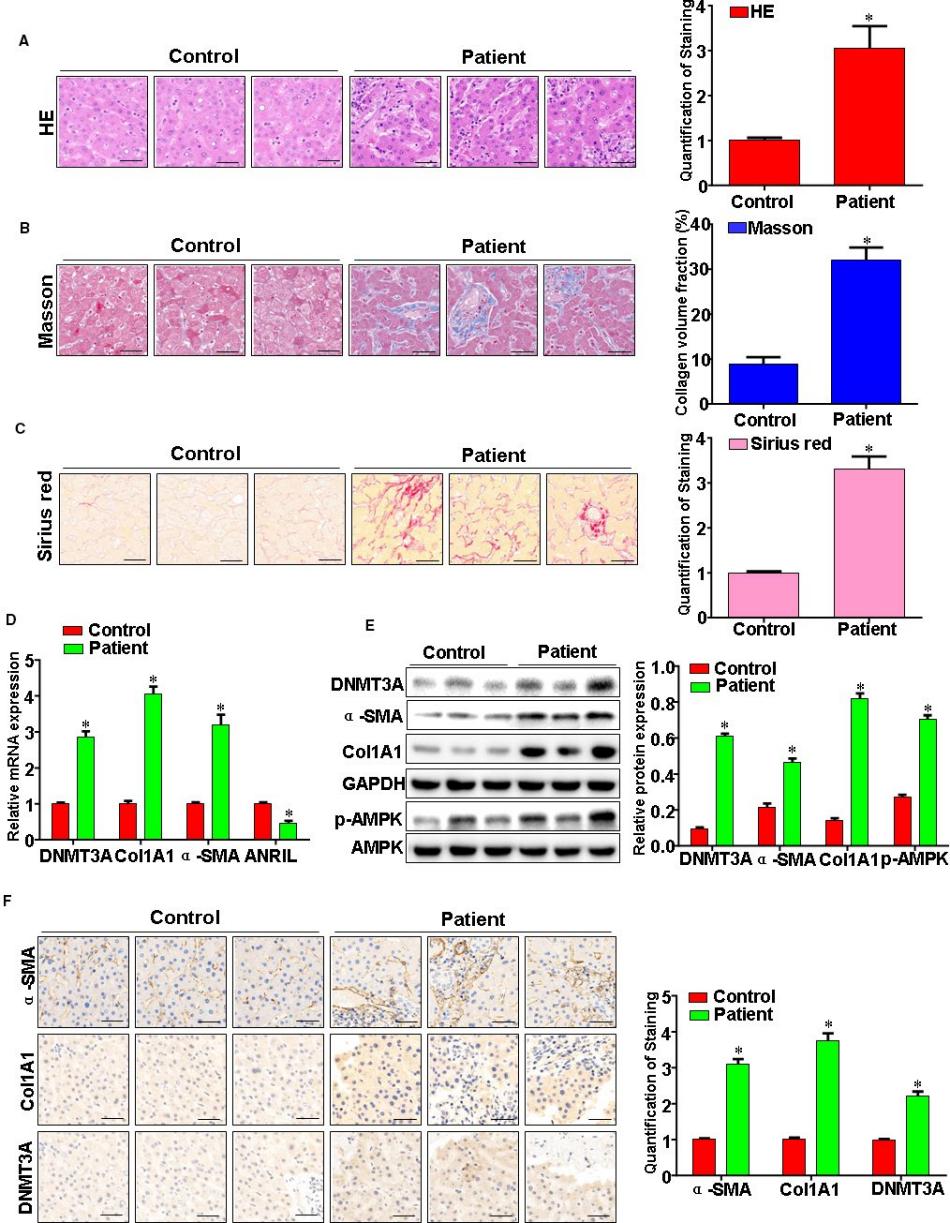
Epigenetic alterations in human chronic liver diseases of distinct aetiologies resemble those associated with rat liver fibrosis. A, Human liver fibrosis tissue was fixed with formalin, and then it was embedded in paraffin. Thin sections were cut and stained with haematoxylin & eosin (H&E), B, Masson's trichrome stain and (C) Sirius red staining. D, DNMT3A, Col1A1, α‐SMA and ANRIL expression were analysed by qRT‐PCR. E, DNMT3A, Col1A1, α‐SMA, AMPK and p‐AMPK expression were analysed by Western blotting. F, The DNMT3A, Col1A1 and α‐SMA expression were evaluated by immunohistochemistry. Data are representative of at least three separate experiments. *P < .05, **P < .01 vs vehicle

We were next interested to discover whether similar epigenetic changes occur in fibrosis human livers. H&E staining found inflammatory infiltration, steatosis and fibrosis in human liver fibrosis tissues (Figure 6A). Moreover, Masson's trichrome staining demonstrated that collagen deposition significantly increased in the human liver fibrosis tissues (Figure 6B). Furthermore, representative stains for Sirius red confirmed in human liver fibrosis tissues (Figure 6C). DNMT3A, α‐SMA, Col1A1 and p‐AMPK were increased in human fibrosis livers, while LncRNA ANRIL was decreased in human fibrosis livers (Figure 6D‐F). These data, collected from mechanistically distinct examples of chronic human liver disease, reveal common fibrosis‐associated epigenetic changes including increased DNMT3A expression, and reduced LncRNA ANRIL that are also observed as characteristic epigenetic features of experimental liver fibrosis in rats.

## 4 DISCUSSION

Hepatic stellate cell activation plays a key role in the pathogenesis of liver fibrosis.^17^ It is known that TGF‐β1 triggers AMPK signal pathway activation in liver fibrosis and HSC activation.^18^ The implication of DNA methylation and LncRNAs in liver fibrosis has been increasingly discovered.^19^, ^20^ In this study, we have demonstrated LncRNA ANRIL, which is involved in the attenuation of HSC activation in liver fibrosis. To our knowledge, this is the first study demonstrating DNMT3A silencing of LncRNA ANRIL regulates HSC activation, thus providing novel mechanistic insights into a critical role for LncRNA ANRIL in liver fibrosis pathogenesis.

LncRNA ANRIL is a recently discovered LncRNA that consistently correlated with fibrosis disease as well as other human diseases like cardiovascular, tumour and so on. Presently, we uncovered significant down‐regulation of LncRNA ANRIL in activated HSC, as well as low expression of this gene in liver fibrosis. We found that LncRNA ANRIL depletion triggers HSC activation. Moreover, LncRNA ANRIL could negatively inhibit the activation of AMPK pathway and the excretion of collagens. In addition, the over expression of LncRNA ANRIL showed an opposite effect in the activated HSC. Interestingly, our results largely implied that LncRNA ANRIL deficient in activated HSC was more likely to be a cellular compensatory mechanism.

There is a growing interest in epigenetic molecular mechanisms for investigations and therapeutic purpose. Aberrant DNMT expressions have been found in liver fibrosis conditions,^21^ but the information regarding specific DNMT subtype involved in liver fibrosis is not appreciatively established. Accumulating evidence indicates the existence of cross regulation between DNA methylation and LncRNAs in the control of gene expression.^22^

DNMT3A was increased in TGF‐β1 induces activated HSC and liver fibrosis. Here, we show the specific role of DNMT3A by means of a targeted knockdown with a siRNA. Knockdown DNMT3A had a profound inhibitory effect on TGF‐β1‐induced HSC proliferation. Moreover, we used 5‐AzadC, which specifically inhibitors the catalytic subunit of DNMT3A. Inhibition of DNMT3A in HSC‐treated 5‐AzadC and TGF‐β1 led to a significant decrease in α‐SMA and Col1A1. We also have been showed that DNMT3A mediates HSC activation in liver fibrosis by down‐regulating the expression of LncRNA ANRIL. At the same time, HSC was treated with 5‐AzadC to promote LncRNA ANRIL expression. We also discovered similar epigenetic changes occur in fibrosis human livers. These data, collected from mechanistically distinct examples of chronic human liver disease, reveal common fibrosis‐associated epigenetic changes including increased DNMT3A expression, and reduced LncRNA ANRIL that are also observed as characteristic epigenetic features of fibrosis human livers.

In conclusion, our study demonstrates that epigenetic silencing of LncRNA ANRIL regulates HSC activation though AMPK pathway is crucial pro‐fibrogenic components forming an epigenetic cascade promoting liver fibrosis. This is a novel understanding of the role of DNMT3A and LncRNA ANRIL in HSC proliferation and the mechanism involved.

## CONFLICT OF INTEREST

The authors declare that there are no conflicts of interest.

## AUTHOR'S CONTRIBUTION

Jing‐Jing Yang wrote the paper; Yang Yang and Chong Zhang participated in the design of the study and performed the statistical analysis, Yan Yang and Jun Li instructed the paper. All authors read and approved the final manuscript.

## Data Availability

The data that support the findings of this study are available from the corresponding author upon reasonable request.

## References

[1] Zou X , Ramachandran P , Kendall TJ , et al. 11Beta‐hydroxysteroid dehydrogenase‐1 deficiency or inhibition enhances hepatic myofibroblast activation in murine liver fibrosis. Hepatology. 2018;67:2167‐2181. 29251794 10.1002/hep.29734 PMC6001805

[2] Shajari S , Laliena A , Heegsma J , Tunon MJ , Moshage H , Faber KN . Melatonin suppresses activation of hepatic stellate cells through RORalpha‐mediated inhibition of 5‐lipoxygenase. J Pineal Res. 2015;59:391‐401. 26308880 10.1111/jpi.12271

[3] Wang Y , Tu K , Liu D , et al. p300 acetyltransferase is a cytoplasm‐to‐nucleus shuttle for SMAD2/3 and TAZ nuclear transport in transforming growth factor beta‐stimulated hepatic stellate cells. Hepatology. 2019;70(4):1409‐1423. 31004519 10.1002/hep.30668 PMC6783326

[4] Moran‐Salvador E , Garcia‐Macia M , Sivaharan A , et al. Fibrogenic activity of MECP2 is regulated by phosphorylation in hepatic stellate cells. Gastroenterology. 2019;157(5):1398‐1412. 31352003 10.1053/j.gastro.2019.07.029 PMC6853276

[5] Jalan‐Sakrikar N , De Assuncao TM , Shi G , et al. Proteasomal degradation of enhancer of zeste homologue 2 in cholangiocytes promotes biliary fibrosis. Hepatology. 2019;70(5):1674‐1689. 31070797 10.1002/hep.30706 PMC6819212

[6] Yang JJ , She Q , Yang Y , Tao H , Li J . DNMT1 controls LncRNA H19/ERK signal pathway in hepatic stellate cell activation and fibrosis. Toxicol Lett. 2018;295:325‐334. 30010033 10.1016/j.toxlet.2018.07.013

[7] Placek K , Schultze JL , Aschenbrenner AC . Epigenetic reprogramming of immune cells in injury, repair, and resolution. J Clin Invest. 2019;130:2994‐3005. 10.1172/JCI124619 PMC6668667 31329166

[8] Kim HG , Huang M , Xin Y , et al. The epigenetic regulator SIRT6 protects the liver from alcohol‐induced tissue injury by reducing oxidative stress in mice. J Hepatol. 2019;71(5):960‐969. 31295533 10.1016/j.jhep.2019.06.019 PMC6801027

[9] Wang M , Zhang K , Ngo V , et al. Identification of DNA motifs that regulate DNA methylation. Nucleic Acids Res. 2019;47:6753‐6768. 31334813 10.1093/nar/gkz483 PMC6649826

[10] Ballarino M , Cipriano A , Tita R , et al. Deficiency in the nuclear long noncoding RNA Charme causes myogenic defects and heart remodeling in mice. EMBO J. 2018;37(18):e99697. 30177572 10.15252/embj.201899697 PMC6138438

[11] Su J , Huang YH , Cui X , et al. Homeobox oncogene activation by pan‐cancer DNA hypermethylation. Genome Biol. 2018;19:108. 30097071 10.1186/s13059-018-1492-3 PMC6085761

[12] Manzo M , Wirz J , Ambrosi C , Villasenor R , Roschitzki B , Baubec T . Isoform‐specific localization of DNMT3A regulates DNA methylation fidelity at bivalent CpG islands. EMBO J. 2017;36:3421‐3434. 29074627 10.15252/embj.201797038 PMC5709737

[13] Gomez L , Odom GJ , Young JI , et al. coMethDMR: accurate identification of co‐methylated and differentially methylated regions in epigenome‐wide association studies with continuous phenotypes. Nucleic Acids Res. 2019;47(17):e98. 31291459 10.1093/nar/gkz590 PMC6753499

[14] Kulkarni S , Lied A , Kulkarni V , et al. CCR5AS lncRNA variation differentially regulates CCR5, influencing HIV disease outcome. Nat Immunol. 2019;20:824‐834. 31209403 10.1038/s41590-019-0406-1 PMC6584055

[15] Kumar A , Thomas SK , Wong KC , et al. Mechanical activation of noncoding‐RNA‐mediated regulation of disease‐associated phenotypes in human cardiomyocytes. Nat Biomed Eng. 2019;3:137‐146. 30911429 10.1038/s41551-018-0344-5 PMC6430136

[16] Wei S , Zhou H , Wang Q , et al. RIP3 deficiency alleviates liver fibrosis by inhibiting ROCK1‐TLR4‐NF‐kappaB pathway in macrophages. FASEB J. 2019;33(10):11180‐11193. 31295018 10.1096/fj.201900752R

[17] Wang S , Kim J , Lee C , et al. Tumor necrosis factor‐inducible gene 6 reprograms hepatic stellate cells into stem‐like cells, which ameliorates liver damage in mouse. Biomaterials. 2019;219:119375. 31374480 10.1016/j.biomaterials.2019.119375

[18] Nguyen G , Park SY , Le CT , Park WS , Choi DH , Cho EH . Metformin ameliorates activation of hepatic stellate cells and hepatic fibrosis by succinate and GPR91 inhibition. Biochem Biophys Res Commun. 2018;495:2649‐2656. 29278707 10.1016/j.bbrc.2017.12.143

[19] Yang Y , Wu XQ , Li WX , et al. PSTPIP2 connects DNA methylation to macrophage polarization in CCL4‐induced mouse model of hepatic fibrosis. Oncogene. 2018;37:6119‐6135. 29993036 10.1038/s41388-018-0383-0

[20] Zhang K , Han Y , Hu Z , et al. SCARNA10, a nuclear‐retained long non‐coding RNA, promotes liver fibrosis and serves as a potential biomarker. Theranostics. 2019;9:3622‐3638. 31281502 10.7150/thno.32935 PMC6587170

[21] Kumar P , Raeman R , Chopyk DM , et al. Adiponectin inhibits hepatic stellate cell activation by targeting the PTEN/AKT pathway. Biochim Biophys Acta. 2018;1864:3537‐3545. 10.1016/j.bbadis.2018.08.012 PMC6529190 30293572

[22] Ponnusamy M , Liu F , Zhang YH , et al. Long noncoding RNA CPR (Cardiomyocyte Proliferation Regulator) regulates cardiomyocyte proliferation and cardiac repair. Circulation. 2019;139:2668‐2684. 30832495 10.1161/CIRCULATIONAHA.118.035832

